# Comparison of Medpor Coated Tear Drainage Tube versus Silicon Tear Drainage Tube in Conjunctivodacryocystorhinostomy: Problems and Solutions

**DOI:** 10.1155/2014/164834

**Published:** 2014-10-14

**Authors:** Selam Yekta Sendul, Halil Huseyin Cagatay, Burcu Dirim, Mehmet Demir, Ali Atakhan Yıldız, Zeynep Acar, Sonmez Cinar, Dilek Guven

**Affiliations:** ^1^Department of Ophthalmology, Sisli Etfal Training and Research Hospital, Halaskargazi Street, Etfal Home Street, Şişli, 34371 Istanbul, Turkey; ^2^Department of Ophthalmology, Faculty of Medicine, Kafkas University, Pasacayiri Street, 36301 Kars, Turkey

## Abstract

*Purpose.* This study aims at comparing two different types of drainage tubes in conjunctivodacryocystorhinostomy, which are used for upper lacrimal system obstruction or damage, with respect to their respective postoperative problems and solutions. *Methods.* Nineteen eyes of 17 patients who underwent conjunctivodacryocystorhinostomy (CDCR) or conjunctivorhinostomy (CR) surgery with a Medpor coated tear drainage tube or silicon tube placement between October, 2010, and February, 2014, were included in this retrospective comparative study. *Results.* In the initial surgery, Medpor coated tear drainage tubes were used in 11 eyes by CDCR, whereas silicon tear drainage tubes were implanted into 2 eyes by CR and 6 eyes by CDCR. In group 1, proximal and distal obstructions developed postoperatively in 4 eyes, while 1 eye showed tube malposition and 3 eyes developed luminal obstruction by debris 3 times. In group 2, tube extrusion developed in 4 eyes, whereas tube malposition developed in 6 eyes and luminal obstruction by debris developed in 6 eyes at different times, for a total of 20 times. *Conclusions.* In our study, the most significant complication we observed in the use of silicon tear drainage tubes was tube extrusion,whereas the leading complication related to the use of Medpor coated tear drainage tubes was tube obstruction.

## 1. Introduction 

Conjunctivodacryocystorhinostomy (CDCR) is a surgical method that involves bypassing the upper lacrimal system and creating a new drainage system between the conjunctiva and the nasal cavity by a tear drainage tube in patients with total upper lacrimal system obstruction or damage. It was first defined by Jones in 1962; there is a tear drainage tube that carries his name [1]. Although CDCR is a standard surgical method, many postoperative complications associated with this procedure such as tube extrusion, malposition and obstruction, and diplopia have been reported. Many patients who underwent this surgery need further surgery or tube repositioning [2, 3]. Many researchers have tried to develop new techniques or tear drainage tube designs in order to reduce complications. Chung et al. [4] used rubber tubes, whereas Trotter and Meyer [5] performed surgery accompanied by nasal endoscopy in order to be able to achieve a more appropriate placement of the tube. Further, Can et al. [6] have covered Jones tubes with buccal mucosa in some cases.

To the best of our knowledge, comparative studies according to the material type used are limited in the literature. In this study, we aimed to assess the etiologies of proximal nasolacrimal system obstruction and compare the results of conjunctivodacryocystorhinostomy (CDCR) and/or conjunctivorhinostomy (CR) performed by two different tear drainage tubes, thereby assessing the relative advantages and disadvantages of each tear drainage tube, postoperative problems, and solutions thereof.

## 2. Material and Method 

Nineteen eyes of 17 patients who underwent conjunctivodacryocystorhinostomy (CDCR) or conjunctivorhinostomy (CR) surgery with a tear drainage tube placement between October 2010 and February 2014 were included in this retrospective comparative study. The patients were divided into two groups. Group 1 consisted of patients in whom Medpor coated tear drainage tubes (Porex Surgical Inc., Newnan, GA, USA) were placed by CDCR or CR, whereas group 2 consisted of patients in whom silicon tear drainage tubes [Metaireau tube (FCI Ophthalmics, Marshfield Hills, MA)] were implanted by CDCR or CR. Those patients with intact middle lacrimal drainage system (lacrimal sac) underwent CDCR surgery, whereas, for those patients with involved middle lacrimal drainage system, CR surgery method was preferred. However, the choice of the tube to be implanted was random. Patients who underwent a follow-up period of less than 3 months and patients who did not regularly show up for follow-ups were excluded. The study was conducted in accordance with the tenets of the Declaration of Helsinki by obtaining written consent from all patients, with the approval of the local ethical review board.

### 2.1. Surgical Technique

**Figure 1:**
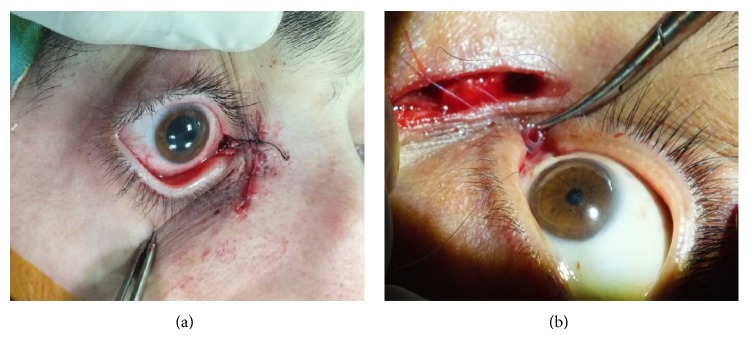
(a) At the final stage of conjunctivodacryocystorhinostomy surgery performed with Medpor coated tear drainage tube, the tube is suspended to the nasal root by a silk suture and then secured. (b) In surgeries in which silicon tear drainage tubes are used, two 7/0 vicryl sutures are passed through the tube valves, thereby securing the tube to conjunctiva and caruncle.

All surgeries were performed under general anesthesia. In the surgical technique, the stages of traditional external dacryocystorhinostomy (EDCR) with H flap were followed for patients who had stable middle lacrimal systems (lacrimal sac). At the flap stage, after the lower flaps (sac and mucosal flap) were sutured, the caruncula was excised in the inner canthus and then a tube bed inclined to the inferior at 30° was opened between conjunctiva and lacrimal sac with a 20-gauge MVR knife. The bed was enlarged and prepared by lacrimal dilatators. Medpor coated tear drainage tubes (18/3.5 mm) were directly passed through the bed, while silicon tubes (40/2 mm) were placed by passing them over lacrimal probes. After the placement of silicon tubes, the distal ends were adjusted and shortened so that they did not touch the nasal septum. After suturing the upper flaps, conventional DCR stages were completed. After that, for securing purposes, 4/0 silk suture was passed through the neck of the proximal end of the Medpor coated drainage tubes, thereby suspending the tube from the nasal root. On the other hand, silicon tubes were sutured to the conjunctiva in the inner canthus by two 7/0 vicryl sutures at the flat proximal end (Figures 1(a) and 1(b)).

All patients were administered 2 × 1000 mg amoxicillin clavulanate potassium daily in postoperative week 1, ciprofloxacin HCL pomade twice a day, and Netildexeye drops (1.32 mg dexamethasone disodium phosphate + 4.55 mg netilmicin sulphate) 4 times a day for 2 weeks. Skin sutures were removed a week later and tube traction sutures a month later. Fixation sutures in the silicon tubes were left to primary dissolution. Follow-ups of the patients were made in postoperative day 1, week 1, month 1, and month 3, and subsequent follow-ups were made semiannually.

Preservation of the drainage tube in its suitable anatomical position and adequate drainage in the postoperative period was used as success criteria.

### 2.2. Statistical Method

In statistics used to describe the data, ratio and frequency values were used. The Chi-square test was used in qualitative analysis; when Chi-square conditions were not met, Fisher's exact test was used. The SPSS 22.0 program was used for analysis.

## 3. Results 

Nineteen eyes of 17 patients were included in the study. The mean age of the patients was 52 ± 17.9 years (range: from 31 to 76). Eleven patients were males (64.7%) and 6 patients were females (35.3%). Nine patients had obstruction in the right eye, 6 patients had obstruction in the left eye, and 2 patients had bilateral obstruction one of whom had bicanalicular obstruction secondary to ichthyosis whereas the other one had bilateral punctual agenesis. Demographics of the study population are summarized in Table 1. The etiological distribution of the patients is summarized in Table 2. CDCR was performed in 17 eyes, whereas CR was performed in 2 eyes (one eye due to upper and middle lacrimal system excision accompanied by tumor excision and one eye due to upper-lower canalicular and lacrimal sac burn after laser DCR). In the initial surgery, Medpor coated tear drainage tubes were implanted by CDCR in 11 eyes, whereas silicon tear drainage tubes were implanted in 2 eyes by CR and 6 eyes by CDCR. The mean follow-up time was 12.88 months (range: from 3 to 40).

### 3.1. Postoperative Complications and Solutions Thereof

#### 3.1.1. Tube Extrusion

After the initial surgery, no tube extrusion developed in patients in group 1, whereas, in group 2, 4 eyes developed tube extrusion. In two of these 4 eyes, the etiology was idiopathic canalicular obstruction, in one eye lacrimal sac excision due to tumor excision and in one eye upper and middle lacrimal system damage secondary to laser DCR; this difference was statistically significant (*P* = 0.029). Tube extrusion was observed in month 1 to month 3. Silicon tubes were placed into two of these patients by CDCR, whereas two of them underwent CR surgery. Two patients who developed tube extrusion were operated on for the second time for implantation of Medpor coated tubes. One of the other two patients refused a second surgery, whereas the last patient in whom the etiology was total canalicular rupture together with middle lacrimal system burn due to laser DCR surgery was not considered for a second surgery due to development of fistulisation.

#### 3.1.2. Obstruction

**Figure 2:**
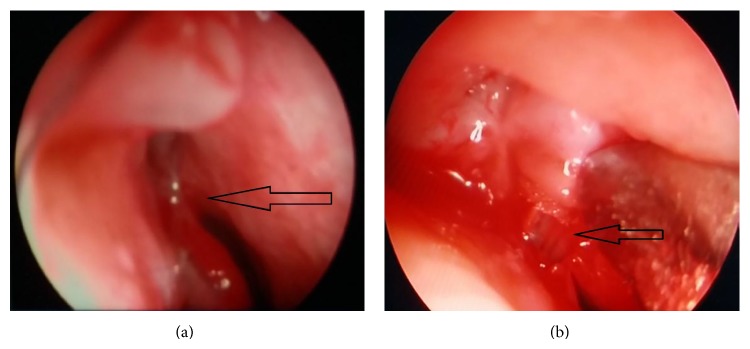
(a) In endoscopic imaging, it is observed that the distal end of the Medpor coated tear drainage tube is completely covered by mucosa and forms synechia with septum (arrow). (b) In the same patient, the mucosal tissue around the tube was excised and the distal end of the tube was revealed (arrow).

After the initial surgery, in group 1, two cases developed proximal obstruction due to conjunctival proliferation, whereas two cases developed distal obstruction and adhesion to septum due to mucosal proliferation. On the other hand, no proximal or distal obstructions developed in any of the cases in group 2. Upon statistical comparison of the two groups, obstruction in group 1 was significantly higher (*P* = 0.031). Both patients who developed proximal obstruction were among our first patients who did not undergo traction by a proximal tube neck suspension suture in the initial surgery. These patients underwent surgical intervention including conjunctival excision, and then traction was applied by passing a silk suture through the proximal neck of the tube. We obtained stabilization of the tubes. Upon these experiences with two patients, we applied traction sutures as routine practice in all patients in whom a Medpor coated tear drainage tube was inserted. One of the patients who developed distal obstruction underwent mucosal excision accompanied by endoscopy, whereas the other case additionally had tube replacement due to the development of conjunctival granuloma together with conjunctival and corneal irritation due to tube malposition (Figures 2(a) and 2(b)).

#### 3.1.3. Lumen Obstruction

Three patients in group 1 developed lumen obstruction for a total of 3 times, due to secretion and debris in the eyes, whereas a total of 20 instances of lumen obstruction were observed at various times in 6 patients in group 2. However, a comparison of the two groups revealed no statistically significant difference (*P* = 0.176). Lumen obstruction was easily overcome by pressure lavage in both groups.

#### 3.1.4. Tube Malposition

**Figure 3:**
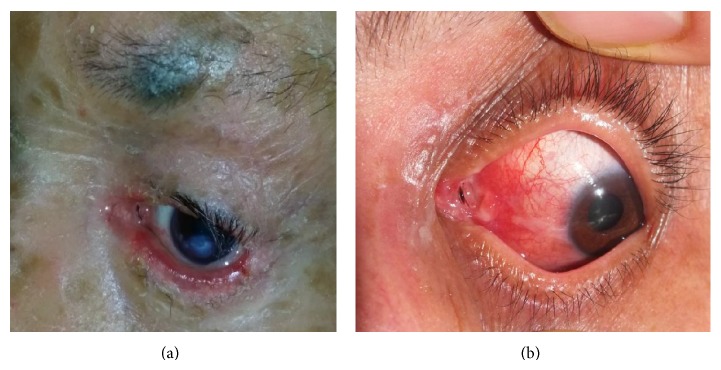
(a) The appearance at the postoperative month 6 of a patient who developed punctum secondary to ichthyosis and canalicular obstruction (the Medpor coated tube is in a suitable position and provides drainage). (b) Fifty-five-year-old female patient developed conjunctival irritation and conjunctival granuloma due to malposition of Medpor coated tube. (Due to ongoing complaints of the case, the tube was replaced).

One patient in group 1 developed tube malposition, whereas 6 patients in group 2; this difference was statistically significant (*P* = 0.015) (Figures 3(a) and 3(b)). One patient in group 2 developed extrusion upon distal shift, while 5 patients developed proximal shift (extrusion in 3 patients). Two of these patients were referred to us in the early period before complete obstruction of the tube bed; therefore, the silicon tubes could be repositioned. Then, they were secured to the conjunctiva by two 7/0 vicryl sutures in the proximal valves of the silicon tubes. As for group 1, since the patient who developed conjunctival granuloma together with conjunctiva and corneal irritation upon tube malposition also developed distal obstruction, reoperation and tube replacement were made. No other complications were observed in either group.

**Figure 4:**
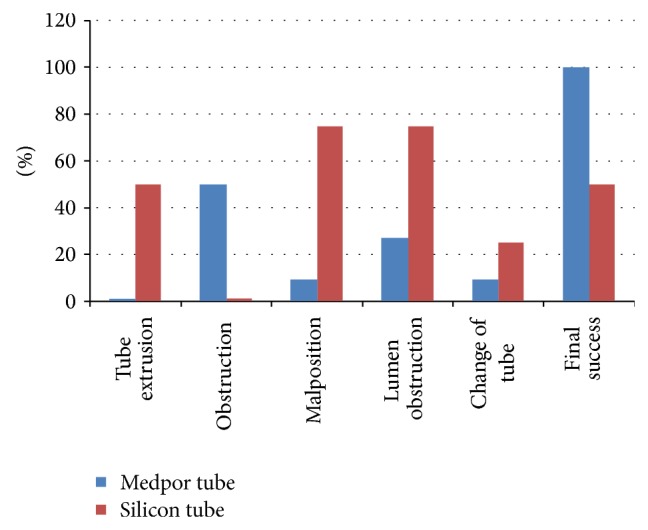
Postoperative complication rates and final success rates of both tubes.

In both patient groups, the number of surgeries and the necessity of the replacement of the tube used in the initial surgery with another one were also compared; no statistically significant results were obtained (*P* = 0.402; *P* = 0.576). The statistical analysis of etiological factors leading to obstruction on different surgical technical results could not be done due to insufficient number of patients. The success rate after initial surgery was 63.63% (7/11) in patients in whom Medpor coated tear drainage tubes were placed, whereas the success rate was 50.00% (4/8) in patients in whom silicon tubes were used. After the second surgery, complete success (100%, *n* = 13) was achieved in all patients with Medpor coated tear drainage tubes. On the other hand, the success rate did not change in patients with silicon tubes due to the fact that Medpor coated tear drainage tubes were preferred in the second surgeries (Figure 4).

## 4. Discussion

Despite various postoperative problems, conjunctivodacryocystorhinostomy with a tear drainage tube placement is a widely accepted surgical method in the treatment of upper nasolacrimal duct obstruction or absence. It may cause complications which may influence surgical success including tube malposition, extrusion, and proximal or distal obstruction as well as relatively milder problems such as conjunctival irritation, corneal abrasion, infection, foreign body sensation in the eye, and lumen obstruction. Various surgical techniques and drainage tube alternatives made of different materials (polypropylene, silicone, teflon, and Pyrex glass) have been tried in order to reduce complications [7–9]. In this study, two different tear drainage tubes, namely, Medpor coated and silicon, were used in CDCR and CR surgeries due to upper nasolacrimal duct obstruction or absence. The superiority of these two types of tubes in comparison to each other, the postoperative complications of both tubes, and our solutions for postoperative complications were evaluated.

The most important complication after the conjunctivodacryocystorhinostomy (CDCR) procedure is obviously tube extrusion, which influences surgical success and usually develops before the formation of a fistula. This usually happens in the postoperative first 6 months [10–12]. The etiology of canalicular obstruction, the surgical method, and the shape and material of the tube used influence tube extrusion [13–16]. Although the most commonly used Pyrex glass tube in the literature provides satisfactory and ideal drainage, its extrusion rate has been reported as 18–51% [2, 12, 17]. Therefore, angular tubes directed to the prevention of tube extrusion, Medpor coated tubes, rubber tubes that prevent shift in the neck part, or tubes that have holes in the proximal end and especially enable preoperative saturation have been developed. Schwarcz et al. [18] in their study in which they compared inferomedial fornix located tubes with conventionally located tubes reported that the choice for inferomedial fornix located tube was more successful. On the other hand, Fan et al. [16], who performed Medpor coated tear drainage tube placement by CDCR, reported that they did not observe tube extrusion in any of the cases. Similarly, our study revealed no tube extrusion in any of the patients in whom Medpor coated tubes were used. On the other hand, tube extrusion at a rate of 50% was observed in the group for whom silicon tubes were used. The fact that the two cases who developed tube extrusion were those patients who underwent CR surgery indicates the question as to whether surgical technique was critical. However, in the second surgery, we performed Medpor coated tube implantation in one of these cases and experienced no postoperative problems. Still, we believe that the question as to whether the CR technique increases the rate of extrusion is an appropriate concern. We believe that the porous coating of Medpor coated tubes provides perfect tissue compatibility, yet their ability to vascularize is a critical hindrance against tube extrusion. On the other hand, the inert structure of silicon tubes does not fuse with the tissue and poses the risk of extrusion not only in the early postoperative period but also at all times.

One of the most important reasons for the failure of CDCR surgery is tube obstruction that forms due to conjunctival or mucosal proliferation, which develops in the postoperative period. Choi and Yang [19] used Pyrex drainage tube implantation in CR surgeries and reported obstruction with tissue proliferation at a rate of 7.1%, whereas Park et al. [20] reported this rate as 7%. Boboridis and Downes [21] reported an obstruction rate of 12.5% in their study with flat, angled, and suture holed Pyrex tubes. On the other hand, Fan et al. [16] reported a higher rate of obstruction (34.6%) compared to previous studies when they used Medpor coated tear drainage tubes. The authors claimed that the reason for this high rate in Medpor coated tubes was unknown, but Medpor may irritate the adjacent mucous membranes and may cause pyogenic granulomas. In our study, an obstruction rate of 36.36% (*n* = 4) developed in the group for whom Medpor coated drainage tubes were used. Two patients developed proximal obstruction after conjunctival proliferation, whereas two patients developed distal obstruction after mucosal proliferation. In both our study and the study by Fan et al. [16], although there are other reasons behind this high rate of obstruction in Medpor coated tubes, obviously the porous structure of Medpor seems to be an important factor. In our view, the causes of this two-sided obstruction may be summarized as follows. (1) Medpor coating is prone to vascularization; especially during the early postoperative period in the phase of surgical cicatrization, synechia develops with Medpor in both the conjunctival and the mucosal scar areas. (2) The fact that the coating on the tube is long increases contact with conjunctival and mucosal surfaces. (3) Standard tube lengths lead to perioperative adjusting difficulties. (4) The separation of the coating part from the glass tube and more shifting to the proximal or distal side during surgical manipulation increase contact with the tissue and cause the development of obstructions. Abdulhafez et al. [22] in their series of 10 cases reported no obstructions in any of the cases and reported complete success by perioperative shortening of the Medpor coating amount. Conjunctival proliferation induced proximal obstruction had developed in our first two cases for whom we used Medpor coated tubes. In these two cases, after excision of the conjunctival tissue around the tube, we suspended it to the nasal root in the inner canthal area by passing 4/0 silk suture around the proximal tube neck; we removed the suspension suture after 1 month. With this modification, we aimed to obtain a space between the proximal end of the tube and the conjunctival tissue until the surgical scar area healed. As a result of the success we achieved in these two cases, we adopted placing a suspension suture as a routine procedure in our surgery and never again experienced proximal obstruction in any of our cases. In any of the cases for whom a silicon tube was used (except extrusion cases), obstructions were not observed as a result of proximal or distal proliferation.

An important problem with this kind of surgery is tube malposition or migration. Malposition or shift of the tube outwards causes ocular surface damage, whereas shifting inwards may lead to pain, obstruction, or mucosal damage [2]. The movement of the tube towards the medial or lateral may lead to situations that necessitate revision, especially during sniffing or coughing [17, 23]. In our study, 6 patients of group 2 showed distal and proximal shift in tubes, whereas only 1 patient in group 1 showed proximal shift. As we sutured patients' silicon tubes to the conjunctiva, shift or malposition in tubes occurred mostly after postoperative month 1 (after the vicryl was sutured). On the other hand, the shift observed in one patient for whom we used a Medpor coated tube occurred in postoperative month 1, and conjunctival granuloma developed due to conjunctival irritation. Shift in silicon tubes may be expected due to their inert structure and stabilization problems, but malposition of a Medpor coated tube was a surprising occurrence because the main advantage of Medpor coated tubes is good stability. We questioned whether we had overenlarged the tube bed during surgery towards the posterior, which was probably so.

One of the important postoperative problems associated with CDCR surgery is intraluminal obstruction due to debris or mucus accumulation. Although it may not require revision, it definitely affects patient comfort. It is not proven but widely considered that the incidence of lumen obstruction is lower in Pyrex tubes than in silicon and polyethylene tubes [24]. Indeed, in our study, lumen obstructions in patients with silicon tube implantation occurred at a much higher rate than in those with Medpor coated tubes, although this difference did not reach statistical significance. We believe that, as the intraluminal lubricity of the glass tube is higher than that of the silicon tube, this leads to lower ground resistance as a result of less obstruction.

## 5. Conclusion 

In this study, we aimed to explore the advantages and disadvantages of two different materials that are used in this surgical technique (Table 3). In our study, the most significant complication observed in the use of silicon tear drainage tubes was tube extrusion, whereas the leading complication in relation to Medpor coated tear drainage tubes was tube obstruction. The method of tube suspension in the postoperative early period that we developed in order to prevent proximal tube obstruction proved to be successful, despite temporary cosmetic visual pollution. Limitations of our study were its retrospective nature and the small number of cases. The small size of the study did not allow us to compare the success rates according to their etiology, and it is known that success rates are likely to improve as the surgeon becomes more experienced with the technique. Further studies with longer follow-up times and a larger number of cases are needed in order to determine certain superiority of the materials.

## Figures and Tables

**Table 1:** Demographic data of patients included in the study.

		Silicon tube		Medpor coated tube		
		*n*	%	*n*	%	*p*
Gender	Female	3	37.5%	4	44.4%	1000
	Male	5	62.5%	5	55.6%	

Side	Bilateral	0	0.0%	2	22.2%	
	Right	1	12.5%	5	55.6%	
	Left	7	87.5%	2	22.2%	

**Table 2:** Various etiologies of conjunctivodacryocystorhinostomy in the study and distribution thereof according to surgical technique (*n*: number of eyes).

Etiology	Silicon tube	Medpor coated tube
	(*n* = 8)	(*n* = 11)
Idiopathic canalicular obstruction	4	7
Punctum agenesis	1	1
Functional obstruction secondary to external DCR	0	1
Canalicular obstruction secondary to ichthyosis	0	2
Traumatic upper lacrimal system damage	1	0
Upper or middle lacrimal system excision secondary to tumor surgery	1	0
Upper or middle lacrimal system damage secondary to multidiode laser DCR	1	0

**Table 3:** The comparative advantages and disadvantages of both tubes.

Medpor coated tube	Silicon tube
Advantages	Advantages
(1) No stabilization problems and perfect tissue compatibility.	(1) Perioperative tube length can be adjusted.
(2) Lower occurrence of lumen obstruction owing to glass lumen internal structure.	(2) It may be used during reoperation.
(3) The ability to vascularize prevents extrusion.	(3) Cheaper.

Disadvantages	Disadvantages
(1) Perioperative adjustment problems due to standard length.	(1) It poses stabilization problems.
(2) Inability to use the coated section in reoperation.	(2) High rates of extrusion and malposition.
(3) Expensive.	(3) High rate of lumen obstruction.
(4) High rates of proximal and distal obstruction in the early postoperative term due to irritation of the adjacent tissues by Medpor coating.	(4) Low success rate.
